# Hearing impairment amongst people with Osteogenesis Imperfecta in Germany

**DOI:** 10.1007/s00405-024-08983-5

**Published:** 2024-09-27

**Authors:** A. Felicio-Briegel, J. Müller, M. Pollotzek, M. Neuling, D. Polterauer, S. Gantner, J. Simon, I. Briegel, F. Simon

**Affiliations:** 1 Department of Otorhinolaryngology, University Hospital, LMU Munich, Marchioninistr. 15, 81377 Munich, Germany; 2 Department of Orthopedics and Trauma Surgery, Musculoskeletal University Center Munich (MUM), University Hospital, LMU Munich, Marchioninistr. 15, 81377 Munich, Germany; 3 Department of Pulmonology, University Hospital, LMU Munich, Marchioninistr. 15, 81377 Munich, Germany

**Keywords:** Osteogenesis imperfecta, Hearing impairment, Hearing loss, Auditory system

## Abstract

**Introduction:**

Hearing impairment concerns a relevant percentage of individuals with Osteogenesis Imperfecta (OI). When looking at the current literature, the percentage of affected individuals with OI varies greatly from 32 to 58% of patients having mild OI and 21% to 27% of patients having moderate to severe OI. Little is known about the German population with OI.

**Method:**

The goal of this study was to detect how many patients with OI, who visited the annual meeting of the German Association for Osteogenesis Imperfecta in 2023, proved to have a hearing impairment. In this prospective, cross-sectional study, each included individual obtained ear microscopy, audiometry, stapedius reflexes, tympanometry, and OAEs. Furthermore, each patient was asked a set of questions concerning the medical history.

**Results:**

Of the included patients, 33% had hearing impairment**.** A significant difference was found for the mean air–bone gap (ABG) as well as the hearing threshold of the right ears. The difference was found between OI type III and IV (p = 0.0127) for the mean ABG and OI type I and IV (p = 0.0138) as well as III and IV (p = 0.0281) for the hearing threshold. Spearman’s rank correlation showed a high correlation between age and hearing threshold. Of the patients between 40 and 50 years old, 56% had hearing loss.

**Conclusion:**

Hearing loss in individuals with OI is still a relevant problem, especially age-related in OI type I. Audiometry should be performed at least when individuals experience subjective hearing loss. The implementation of a screening starting at 40 years should be discussed and studied.

## Introduction

Osteogenesis Imperfecta (OI) is a group of genetic diseases in which the synthesis of the bone matrix is affected. The latter results in fragile bones with minor trauma causing the bones to break. The clinical severity varies from intrauterine lethality to low fracture incidence. Genetically 22 different types are differentiated [1]. Forlino et al. offers a classification that takes genetical information into account [2]. In Table 1 type of OI, appearance and form are specified.Most cases (> 80%) have autosomal-dominant heterozygote Mutation in Genes of collagensythesis (COL1A1 or COL1A2) [3]. In Germany an approximate of 4000 people are affected by OI [4].

**Table 1:** OI types and genetics [1–3]

OI type by Forlino (2)	Genetic mutation	Form	Type of defect
I	COL1A1 or COL1A2	Mild	Defects in collagen, synthesie, structure, or processing
II	COL1A1 or COL1A2	Lethal	
III	COL1A1 or COL1A2	Severe	
IV	COL1A1 or COL1A2	Mild to moderate	
V	IFITM5	Moderate to severe	Defects in bone mineralization
VI	SERPINF1	Moderate to severe	
VII	CRTAP	Moderate	Defects in collagen modification
VIII	LEPRE1/ P3H1	Lethal or severe	
IX	PP1B	Lethal or severe	
X	SERPINH1	Lethal or severe	Defects in collagen folding and cross-linking
XI	FKBP10	Severe	
XII	SP7	Moderate to severe	Defects in osteoblast development with collagen insufficiency
XIII	BMP1	Moderate to severe	Defects in collagen, synthesie, structure, or processing
XIV	TMEM38B	Moderate to severe	Defects in collagen modification
XV	WNT1	Moderate to severe	Defects in osteoblast development with collagen insufficiency
XVI	CREB3L1	Severe	
XVII	SPARC/Osteonectin	Severe	
XVIII	MBTPS2/S2P	Severe	
XIX	TENT5A/FAM46A	Severe	Defects in collagen, synthesie, structure, or processing
XX	MESD	Lethal or severe	Defect in bone mineralization
XXI	KDELR2	Severe	Defect in collagen transport
XXII	*CCDC134*	Severe	Defects in bone mineralization

Hearing impairment concerns a relevant percentage of individuals with Osteogenesis Imperfecta (OI). Depending on the literature, 32–58% of patients have mild OI, and 21–27% of patients have moderate to severe OI [5, 6]. Conductive and sensorineural, as well as mixed hearing loss can be found. Conductive hearing loss can mainly be found in younger patients with OI type IV, whereas sensorineural and mixed hearing loss concern mainly older patients with OI type I according to Sillence et al. [7, 8]. Conductive hearing loss is thought to be caused by stapes fracture, and bone remodeling with columnar stapes with or without fixation of the footplate [5, 6]. Sensorineural hearing loss is thought to be caused by microfractures of the cochlea, retro-cochlear otosclerosis, and atrophy of the stria vascularis and hair cells. In children and young adolescents with OI, a broad variety of data ranging from 22 to 31% of the 4–9 years old and 28–62% of the 10 to 19 years old to 4.4% of the 4–16 years old exists .

Treatment options for conductive hearing loss include hearing aids and established surgery techniques for middle ear surgery. When sensorineural hearing loss has reached a certain degree and cannot be treated sufficiently by hearing aids, cochlea implantation is a possible therapeutic option with a good results. Untreated hearing loss can have a negative impact on children, young adults, and adults. Untreated presbycusis for example, can negatively impact cognitive, emotional, and social behavior [9]. Perception of one’s language can be negatively impaired [10]. In children, delayed speech development can be seen in children with middle to high impairment of hearing function in both ears without treatment [11]. Hearing impairment in one ear or mild hearing impairment in both ears can lead to hearing associated fatigue [12].

Until now recurrent audiometry does not belong to standard follow-up of patients with OI. Hearing tests are conducted only when patients report subjective hearing impairment, which can delay treatment. The aim of this study was to determine the prevalence of hearing impairment among patients with OI who attended the annual meeting of the German Association for Osteogenesis Imperfecta.

## Methods

During the 2023 annual meeting of the German Association for Oteogenesis Imperfecta, 39 patients with OI were included in this prospective, cross-sectional study. 23 patients were female and 16 were male. The distribution of OI types was as follows: Ten patients had OI type I, 18 patients had OI type III, 9 patients had OI type IV, and 2 patients had OI type V. Participation in the study was voluntary. Each included individual obtained ear microscopy using “ATMOS i View 21” (ATMOS MedizinTechnik GmbH&Co. KG; Lenzkirch; Deutschland). Furthernore audiometry, stapdius reflexes, and tympanometry was performed using “ATMOS Audio Cube 31” (ATMOS MedizinTechnik GmbH&Co. KG; Lenzkirch; Deutschland). OAE measurements was done using Sentiero (Path Medical Solutions, Germany). Furthermore, each patient was asked a set of questions concerning the medical history: pre-existing illnesses and surgeries concerning the ear and hearing, previous and current medication for OI, previous investigations of the ear and hearing, as well as type of OI. Patient demographics can be found in Table 1.

For the type of OI, the classification by Forlino et al. was used [2]. This classification is based on the classification by David Sillence [8] but also takes into account the genotype, resulting initially in a total of 18 types. 4 types have been added by Jovanovic et al. recently [1].

During pure tone audiometry, the frequencies 250 Hz, 500 Hz, 1 kHz, 2 kHz, 4 kHz, and 6 kHz were tested. Audiometry results were classified as normal if the hearing threshold was lower than 30 dB and pathologic if the threshold was 30 dB or higher in one or more frequencies. The mean hearing threshold was deducted from the acquired data as well as the mean air-bone-gap (ABG) and the mean hearing threshold of bone conduction. Tympanometry was performed using a probe tone of 226 Hz. Stapedius reflexes were measured at 500 Hz, 1 kHz, 2 kHz, and 4 kHz. TEOAE screening was measured and DPOAEs were measured for 1 kHz, 2 kHz, 4 kHz, and 6 kHz. Audiometry was performed in accordance to German standard of procedure for clinical trials [13].

Calculations were performed with R (version 4.3.1, The R Foundation for Statistical Computing, Vienna, Austria). Wilcoxon test for paired groups, Spearman’s rank correlation and Kruskal–Wallis test followed by Dunns’ test if significant were performed.

## Results

The median age of the participants was 33 years (y) with a mean age of 37 y (standard deviation (sd) 16 y, min 6 y, max 69 y). The mean age by the OI group was 50 y for OI type I, 32 y for OI type III, 34 y for OI type IV, and 22 y for OI type V. The median hearing threshold for the right ear was 21 dB ( mean: 30 (sd 28 dB) and for the left ear the median was 15 dB and mean 28 dB (sd 26 dB). The median ABG was 11 dB for the right ear with mean being 7 dB (sd 11 dB). For the left ear median was 6 dB and mean 10 dB (sd 11 dB). One patient had received cochlea implant. Two patients had undergone middle ear surgery having been diagnosed with otosclerosis and one patient had received Tympanoplastik due to luxation of the middle ear ossicles. Four patients had received hearing aids due to hearing loss. Two patients had a family history of hearing loss. One patient had experienced noise trauma. One patient had experienced reccurent middle ear infection as a child. Three patient had experienced subjective hearing loss after head trauma.

**Fig. 1:**
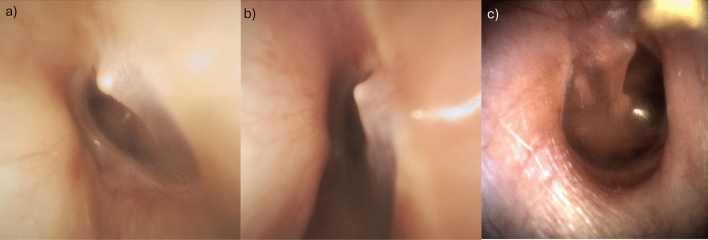
The eardrum of two individuals with OI type III (sub-Figs. **a** and **b**) as well as of one individual with OI type I (sub-Fig. **c**)

Regarding ear microscopy for OI type III, the anterior tympano-meatal angle was found to be far more acute than in healthy or phenotypically not affected OI type I individuals, as shown in Fig. 1.

All patients with moderate or profound hearing loss had received therapy, though all the patients were referred to a larger hospital for the implementation of the therapy. None of the patients had used or tested hearing aids. Further results can be found in Table 2.

**Table 2:** Patients demographics

	OI type I	OI type III	OI type IV	OI type V
Number of included ears	20	36	18	4
Conductive hearing loss [ears]	0	3	0	0
Mixed hearing loss [ears]	5	9	0	0
Sensorineural hearing loss [ears]	7	2	0	0
TEOAE [ears]	Pass: 8 Refer: 12	Pass: 20 Refer: 16	Pass: 16 Refer: 2	Pass: 4 Refer: 0
Stapediusreflexes 0.5 kHz [ears]	Pass: 6 Refer: 12 n/a: 2	Pass: 17 Refer: 18 n/a: 1	Pass: 4 Refer: 14	Pass: 0 Refer: 4
Stapediusreflexes 4 kHz [ears]	Pass: 5 Refer: 13 n/a: 2	Pass: 5 Refer: 29 n/a: 2	Pass: 4 Refer: 14	Pass: 0 Refer: 4
Mean ABG right/left ear	Right: 11 dB Left: 10 dB	Right: 16 dB Left: 13 dB	Right: 5 dB Left: 5 dB	Right: 5 dB Left: 5 dB
Mean hearing threshold right/left ear	Right: 42 dB Left: 44 dB	Right: 34 dB Left 28 dB	Right: 13 dB Left: 15 dB	Right: 12 dB Left 11 dB
Mean ear canal volume	Right:1,32 ml Left: 1,39 ml	Right: 1,18 ml Left: 1,11 ml	Right: 0,95 ml Left: 0,92 ml	Right: 0,96 ml Left: 0,95 ml
Tympanometry[ears]	Type A: 20	Type A: 34 Type As: 2	Type A: 16 Type B: 2	Type A: 4

First mean ABG als well as mean hearing threshold by ear were compared one to another via the Wilcoxon test for paired groups. No statistically significant difference was found.

**Fig. 2:**
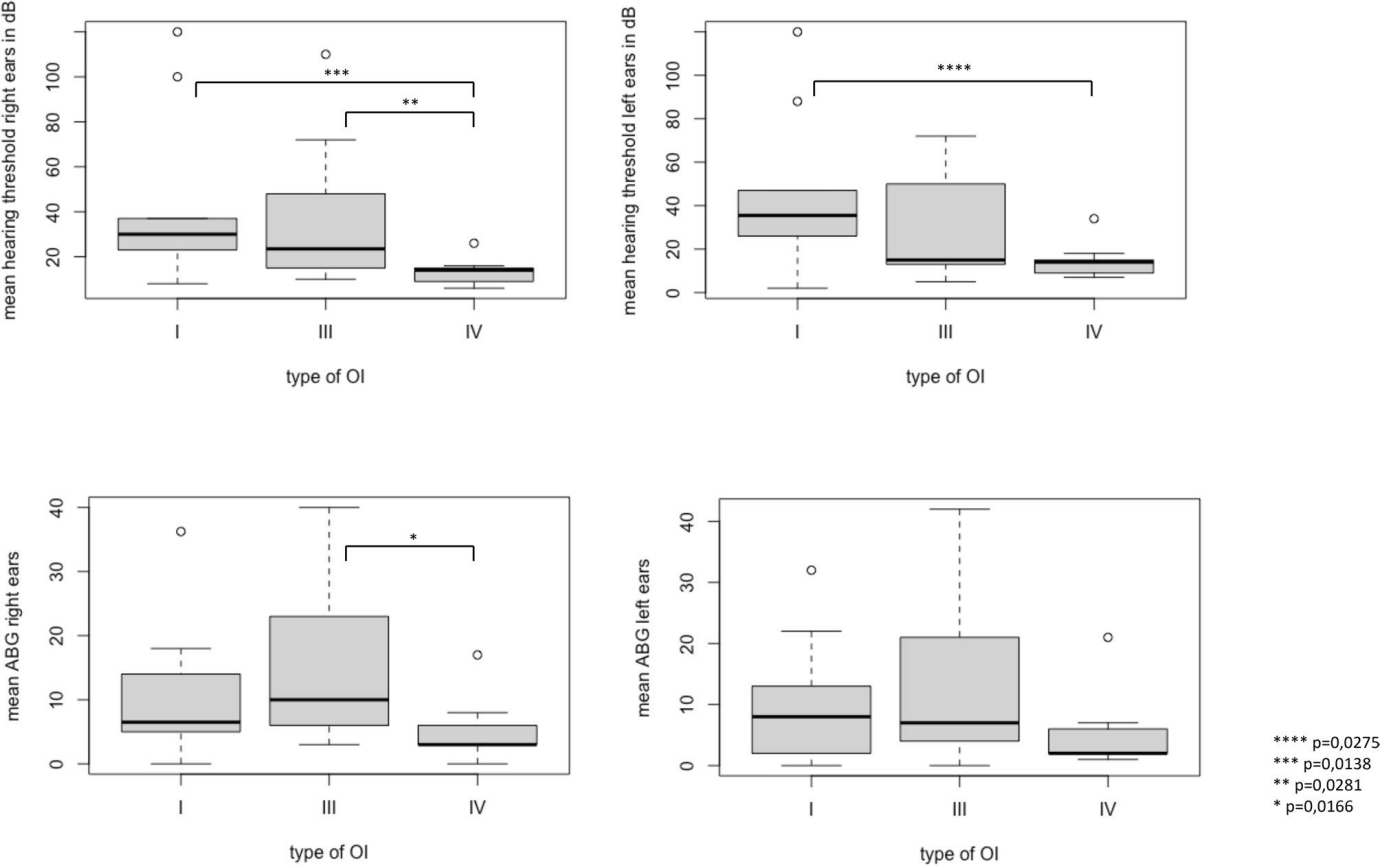
Boxplot of hearing threshold and mean ABG by type of OI. Explanation of boxes: thick black line = median; box boundaries = 25th and 75th percentiles; whiskers = minimum and maximum; circles = outliers. OI = Osteogenesis Imperfecta; dB = decibel; ABG = air-bone-gap; * = significant difference

Mean ABG and hearing threshold between types of OI were compared using the Kruskal–Wallis test. Patients with OI type V were excluded for those tests due to the low number in comparison to the other groups. A significant difference was found for mean ABG the right ears (p = 0.0166), Dunns’ test revealed a significant difference between OI type III and IV (p = 0.0127). In contrast, no significant difference was found for left ears (p = 0.0181). For hearing thresholds, a significant difference was found for the right ears (p = 0.0094). Dunns’ test showed a significant difference between groups of OI I and IV (p = 0.0138) and between III and IV (p = 0.0281). Left ears were also significant (p = 0.0275). Dunns’ test revealed a significant difference between groups of OI I and IV (p = 0.0241). A Boxplot of the mean hearing threshold and ABG is shown in Fig. 2.

Ear canal volume was compared using the Kruskal–Wallis test and was found to be significant for left ears (p = 0.0203). Dunns’ test revealed a significant difference between OI type I and IV (p = 0.0158).

**Fig. 3:**
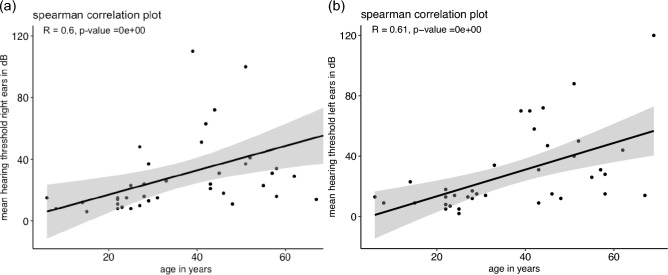
Spearman correlation plot for mean hearing threshold of right ears (subfigure **a**) and left ears (subfigure **b**) by age. Rho and p are specified underneath the title

Spearman’s rank correlation showed a positive and high correlation between age and hearing threshold for both right and left ears (right: rho = 0.0598, p = 0.00003; left: rho = 0.0612, p = 0.00002) as seen in Fig. 3. Bone conduction hearing threshold showed a positive and high correlation with age for right and left ears (right: rho = 0.6435, p = 0.000005; left: rho = 0.6308, p = 0.000008). The hearing threshold of the right and left ears also showed positive and high correlation (rho = 0.8107, p = 0.0000000002). When analyzing the percentage of patients affected by hearing impairment by age, no patient under 20 years had hearing loss. From 20 to 30 years, 7% of the included patients had hearing loss. All of them had conductive hearing loss. Among the patients between 30 to 40 years, 33% had hearing loss. Between 40 and 50 years, 56% had hearing loss. In those two groups, none of the patients had conductive hearing loss. Of the patients older than 50 years, 65% had hearing loss. Only one patient had conductive hearing loss. 69% of the patients in the group older than 50 years had OI type I.

## Discussion

A few studies exist regarding hearing loss in patients with OI, though most of them were published around the 2000-year mark [6, 14–16]. No cross-sectional study regarding the hearing abilities of the German population with OI was found. Furthermore, when acquiring hearing data, one difficulty is, that most patients interested experienced some kind of subjective hearing loss [5]. Machol suggested this selection bias concerning some early studies regarding Osteogenesis Imperfecta and hearing. To avoid such a bias, sample acquirement was done during the annual meeting of the German Association for Osteogenesis Imperfecta. Most participants with OI were included in this study.

Kuurila et al. found hearing loss in half of the included population. The mean age of the participants was 52 years [6]. In this study, hearing loss was found in 33% of the patients. This can be related to the lower mean age in this study, which was 33 years. Pillion et al. as well as Hartikka et al. found 62–65% of hearing loss despite a similarly low mean age. This could be due to the difference in the definition of hearing loss. The latter was defined as a hearing threshold equal to or lower than 15 dB under 60 years and 20 dB over 60 years. In this study, a pathologic hearing threshold was defined as 30 dB or higher. This was chosen in alignment with the definition of a pathologic audiometry in Germany and in alignment with the HORSTAT study [17]. A hearing threshold with 30 dB or worse would in many cases lead to the recommendation of a therapy. Furthermore, such a hearing threshold can be verified by TEOAEs in cases of unclarity. TEOAE are also used to screen for hearing impairment in newborns. Positive TEOAE, which are positive up to a hearing threshold of 25 dB, are thought to exclude a hearing impairment in need of therapy [18]. In alignment with our findings, Waissbluth found hearing loss in 30% of the included patients with a median age of 20 years [19]. The rate of hearing loss of 33% in this study is higher than in the general German population, where the rate of hearing loss was found to be 16% [17]. This is in line with the findings of other countries, where the rate of hearing loss in the general population is around 10% [20]. Hearing loss is thus higher in patients with Osteogenesis Imperfecta.

Similarly to Kuurila et al., mainly mixed and sensorineural hearing loss was found in patients with OI type I, and conductive and mixed hearing loss was found in patients with OI type III [6]. For type IV and V OI, none of the included patients had hearing loss. For OI type V this can be due to the small patient number. Machol et al. also found age-related sensorineural hearing loss mainly in patients with OI type I. For OI type IV, they found only conductive hearing loss [5]. Other studies have also found mixed hearing loss to be the most dominant type of hearing loss, with conductive hearing loss mainly found in younger patients and an increasing sensorineural hearing loss component in older patients [21, 22]. Studies inquiring about hearing loss by type of OI are sparse.

This study also found a positive correlation between the hearing threshold of the left and right ear, indicating, that a patient with hearing loss in one ear, is highly likely to have a hearing loss on the other side [6]. In our study hearing loss was found to start earlier than presbycusis. Presbycusis is thought to start at 60 years [17]. In this study, beginning at 30 years the percentage of patients with hearing loss is higher than the average hearing loss in the general population with the percentage of OI patients with hearing impairment being 33% and in the general population around 10–16% [17, 20]. Similarly to other findings hearing loss formed a plateau around 40 years [7, 23–25]. Almost two-thirds of the patients with OI over 40 years old have hearing loss. This is an even higher percentage than the rate of presbycusis, where 40% of patients older than 65 years have hearing loss [20]. Hearing screenings are discussed for older adults. Positive effects of hearing screening in older adults have been found, for example, hearing screening was found to lead to significantly more hearing aid use [26]. Furthermore, evidence exists regarding the positive cost-effectiveness of hearing screening in older adults [27]. Hearing screening for persons with Osteogenesis Imperfecta beginning at the age of 40 years should be discussed.

Though all patients with moderate to profound hearing loss in this study received treatment, it has to be noted, that all patients were transferred to a bigger hospital for initialization of the therapy. This is usually associated with a delayed start of the therapy due to longer waiting times for an appointment in the hospital. Untreated presbycusis loss is known to contribute to cognitive decline [9, 28]. Hearing screening could positively influence the time between the onset of the hearing loss and the start of the therapy. For instance, patients with mild hearing loss could already be scheduled for presentation in a larger hospital.

## Conclusion

Hearing loss in individuals with Osteogenesis Imperfecta is still a relevant problem, especially age-related in Osteogenesis Imperfecta type I. Audiometry should be performed at least when individuals experience subjective hearing loss. The implementation of a screening starting at 40 years should be discussed and studied. Furthermore, to enable better patient-centered care of people with Osteogenesis Imperfecta it is crucial to provide sufficient information about medical centers that perform middle ear surgeries on OI patients.

## Data Availability

The data presented in this study are available on request from the corresponding authors. The data are not publicly available due to restrictions of the institutional IRB statement in concordance to European/German legislation on data restriction.

## References

[1] Jovanovic M, Marini JC (2024) Update on the Genetics of Osteogenesis Imperfecta. Calcif Tissue Int 10.1007/s00223-024-01266-5 PMC11607015 39127989

[2] Forlino A, Marini JC (2016) Osteogenesis imperfecta. Lancet 387(10028):1657–1671 26542481 10.1016/S0140-6736(15)00728-X PMC7384887

[3] Marini JC, Forlino A, Bachinger HP, Bishop NJ, Byers PH, Paepe A et al (2017) Osteogenesis imperfecta Nat Rev Dis Primers 3:17052 28820180 10.1038/nrdp.2017.52

[4] Hoyer-Kuhn JB-SH, Blickheuser R, Deimling U, Stücker R, Wirth T, Wolf J, Wollinsky KH & Semler O (2016) Diagnostik und Therapie der Osteogenesis imperfecta - Konsensus-Statement der 30. Jahrestagung 2014 der Deutschen Gesellschaft für Osteogenesis imperfecta Betroffene e.V. Monatsschrift Kinderheilkunde. 165:333–346

[5] Machol K, Hadley TD, Schmidt J, Cuthbertson D, Traboulsi H, Silva RC et al (2020) Hearing loss in individuals with osteogenesis imperfecta in North America: results from a multicenter study. Am J Med Genet A 182(4):697–704 31876392 10.1002/ajmg.a.61464 PMC7385724

[6] Kuurila K, Kaitila I, Johansson R, Grenman R (2002) Hearing loss in Finnish adults with osteogenesis imperfecta: a nationwide survey. Ann Otol Rhinol Laryngol 111(10):939–946 12389865 10.1177/000348940211101014

[7] Carre F, Achard S, Rouillon I, Parodi M, Loundon N (2019) Hearing impairment and osteogenesis imperfecta: literature review. Eur Ann Otorhinolaryngol Head Neck Dis 136(5):379–383 31202667 10.1016/j.anorl.2019.05.004

[8] Sillence DO, Senn A, Danks DM (1979) Genetic heterogeneity in osteogenesis imperfecta. J Med Genet 16(2):101–116 458828 10.1136/jmg.16.2.101 PMC1012733

[9] Fischer N, Weber B, Riechelmann H (2016) Presbycusis - age related hearing loss. Laryngorhinootologie 95(7):497–510 27392191 10.1055/s-0042-106918

[10] Hengen J, Hammarstrom IL, Stenfelt S (2018) Perceived voice quality and voice-related problems among older adults with hearing impairments. J Speech Lang Hear Res 61(9):2168–2178 30167670 10.1044/2018_JSLHR-S-17-0383

[11] Moeller MP, McCleary E, Putman C, Tyler-Krings A, Hoover B, Stelmachowicz P (2010) Longitudinal development of phonology and morphology in children with late-identified mild-moderate sensorineural hearing loss. Ear Hear 31(5):625–635 20548239 10.1097/AUD.0b013e3181df5cc2 PMC2932864

[12] Bess FH, Davis H, Camarata S, Hornsby BWY (2020) Listening-related fatigue in children with unilateral hearing loss. Lang Speech Hear Serv Sch 51(1):84–97 31913803 10.1044/2019_LSHSS-OCHL-19-0017 PMC7251590

[13] Rahne T, Dziemba O, Lodwig A, Polterauer D, Thie R, Walger M, et al (2019) ADANO recommendations for the selection of target parameters and measurement processes for the use of auditory evoked potentials, otoacoustic emissions, and impedance audiometry in clinical trials : prepared by the ERA consortium (AG-ERA)(*) of ADANO(#). Confirmed by the board of ADANO on 18.01.2019. HNO 67(Suppl 2):59–61 10.1007/s00106-019-0647-1 31119331

[14] Kuurila K, Grenman R, Johansson R, Kaitila I (2000) Hearing loss in children with osteogenesis imperfecta. Eur J Pediatr 159(7):515–519 10923226 10.1007/s004310051322

[15] Pillion JP, Shapiro J (2008) Audiological findings in osteogenesis imperfecta. J Am Acad Audiol 19(8):595–601 19323351 10.3766/jaaa.19.8.3

[16] Paterson CR, Monk EA, McAllion SJ (2001) How common is hearing impairment in osteogenesis imperfecta? J Laryngol Otol 115(4):280–282 11276328 10.1258/0022215011907442

[17] von Gablenz P, Holube I (2015) Prevalence of hearing impairment in northwestern Germany. Results of an epidemiological study on hearing status (HORSTAT). HNO. 63(3):195–214 25720301 10.1007/s00106-014-2949-7

[18] Brockow I, Sohl K, Hanauer M, Heissenhuber A, Marzi C, Am Zehnhoff-Dinnesen A et al (2023) Newborn hearing screening in Germany-results of the 2011/2012 and 2017/2018 evaluations. Bundesgesundheitsblatt Gesundheitsforschung Gesundheitsschutz 66(11):1259–1267 37843595 10.1007/s00103-023-03779-0 PMC10622351

[19] Waissbluth S, Lira K, Aracena K, Oyarzun J, Willson M, Seiltgens C (2020) Observed frequency and characteristics of hearing loss in osteogenesis imperfecta. Rev Med Chil 148(12):1781–1786 33844744 10.4067/S0034-98872020001201781

[20] Ries PW (1994) Prevalence and characteristics of persons with hearing trouble: United States, 1990–91. Vital Health Stat 10(188):1–75 8165784

[21] Swinnen FK, Dhooge IJ, Coucke PJ, D’Eufemia P, Zardo F, Garretsen TJ et al (2012) Audiologic phenotype of osteogenesis imperfecta: use in clinical differentiation. Otol Neurotol 33(2):115–122 22143304 10.1097/MAO.0b013e31823e28e9

[22] Hartikka H, Kuurila K, Korkko J, Kaitila I, Grenman R, Pynnonen S et al (2004) Lack of correlation between the type of COL1A1 or COL1A2 mutation and hearing loss in osteogenesis imperfecta patients. Hum Mutat 24(2):147–154 15241796 10.1002/humu.20071

[23] Garretsen AJ, Cremers CW, Huygen PL (1997) Hearing loss (in nonoperated ears) in relation to age in osteogenesis imperfecta type I. Ann Otol Rhinol Laryngol 106(7 Pt 1):575–582 9228859 10.1177/000348949710600709

[24] Pedersen U (1984) Hearing loss in patients with osteogenesis imperfecta. A clinical and audiological study of 201 patients. Scand Audiol. 13(2):67–74 6463554 10.3109/01050398409043042

[25] Sillence DO (1988) Osteogenesis imperfecta nosology and genetics. Ann N Y Acad Sci 543:1–15 3063156 10.1111/j.1749-6632.1988.tb55311.x

[26] Yueh B, Collins MP, Souza PE, Boyko EJ, Loovis CF, Heagerty PJ et al (2010) Long-term effectiveness of screening for hearing loss: the screening for auditory impairment–which hearing assessment test (SAI-WHAT) randomized trial. J Am Geriatr Soc 58(3):427–434 20398111 10.1111/j.1532-5415.2010.02738.x

[27] Hsu AK, Bassett SM, O’Dwyer LC, McHugh M, Heinemann AW, Jordan N et al (2022) Cost-effectiveness of hearing screening in older adults: a scoping review. Res Aging 44(2):186–204 33973495 10.1177/01640275211008583

[28] Loughrey DG, Kelly ME, Kelley GA, Brennan S, Lawlor BA (2018) Association of age-related hearing loss with cognitive function, cognitive impairment, and dementia: a systematic review and meta-analysis. JAMA Otolaryngol Head Neck Surg 144(2):115–126 29222544 10.1001/jamaoto.2017.2513 PMC5824986

